# A high altitude respiration and SpO2 dataset for assessing the human response to hypoxia

**DOI:** 10.1038/s41597-024-03065-x

**Published:** 2024-02-27

**Authors:** Xi Zhang, Yu Zhang, Yingjun Si, Nan Gao, Honghao Zhang, Hui Yang

**Affiliations:** 1 School of Life Sciences, Northwestern Polytechnical University, Xi’an, 710072 China; 2 Engineering Research Center of Chinese Ministry of Education for Biological Diagnosis, Treatment and Protection Technology and Equipment, Northwestern Polytechnical University, Xi’an, 710072 China; 3 School of Computer Science, Northwestern Polytechnical University, Xi’an, 710129 China; 4 Department of Computer Science and Technology, Tsinghua University, Beijing, 100084 China; 5 School of Mechanical Engineering, Northwestern Polytechnical University, Xi’an, 710072 China

**Keywords:** Risk factors, Predictive markers, Hypoxia, Diagnostic markers

## Abstract

This report presents the Harespod dataset, an open dataset for high altitude hypoxia research, which includes respiration and SpO2 data. The dataset was collected from 15 college students aged 23–31 in a hypobaric oxygen chamber, during simulated altitude changes and induced hypoxia. Real-time physiological data, such as oxygen saturation waveforms, oxygen saturation, respiratory waveforms, heart rate, and pulse rate, were obtained at 100 Hz. Approximately 12 hours of valid data were collected from all participants. Researchers can easily identify the altitude corresponding to physiological signals based on their inherent patterns. Time markers were also recorded during altitude changes to facilitate realistic annotation of physiological signals and analysis of time-difference-of-arrival between various physiological signals for the same altitude change event. In high altitude scenarios, this dataset can be used to enhance the detection of human hypoxia states, predict respiratory waveforms, and develop related hardware devices. It will serve as a valuable and standardized resource for researchers in the field of high altitude hypoxia research, enabling comprehensive analysis and comparison.

## Background & Summary

Plateaus are common geographical features that cover a significant portion of the Earth’s land surface, accounting for nearly 45% of it^1^. By utilizing the Global Digital Elevation Map provided by NASA Worldview (https://worldview.earthdata.nasa.gov/), we have discovered that plateaus above 2 *km* in altitude are primarily located in western North America, western South America, central Asia, the Middle East, and eastern Africa. However, entering a plateau without undergoing long-term adaptive training can be perilous. Mild altitude sickness may cause individuals to experience shortness of breath, chest tightness, and headaches. In severe cases, inadequate oxygen levels in the bloodstream can result in pulmonary and cerebral syndromes^2–4^, posing a threat to an individual’s life. The most expedient and effective solution in such situations is the provision of sufficient oxygen. Nonetheless, the availability of oxygen is generally limited, especially when considering mobile oxygen supply. The farther individuals are from base camp, the greater the danger they face. Therefore, it is crucial to accurately assess their hypoxia state in order to determine their actual oxygen requirements. This determination not only aids in providing the necessary oxygen but also helps prevent severe altitude sickness. Additionally, it assists in reducing the risk of life-threatening conditions and enhancing the safety of individuals visiting high altitude areas.

The selection of monitoring indicators and the influence of environmental factors are both crucial. Oxygen saturation and respiration rate are crucial indicators for hypoxia. The human respiratory and circulatory systems can indirectly reflect varying degrees of hypoxia through various physiological indicators^5,6^. Clinical assessment of hypoxia in the human body commonly relies on oxygen saturation levels^7,8^. Respiratory patterns not only respond to hypoxia^9,10^, but also have the ability to counteract it. Therefore, real-time assessment of the body’s hypoxic state can be achieved by gathering data on respiration and oxygen saturation. However, the current models used for physiological signal analysis are constructed based on data from plain or pathological scenarios and may not be applicable to plateau environments due to low pressure and hypoxic factors. The greater variability in the pattern of physiological waveforms^11–15^ caused by these factors can hinder the accurate identification of a person’s hypoxic state. In comparison, the time differences between physiological signals^16–21^ on the plains have a relatively constant range. The hypoxic factors of the plateau environment cause changing patterns in time shift that vary greatly between individuals, which can mislead the models and lead to incorrect predictions. These limitations contribute to the lack of applicability of existing models to plateau scenarios. When dealing with prediction tasks in plateau scenarios, using incorrect parameters and strategies can be fatal.

The primary obstacle in the field of high-altitude physiology research is the lack of suitable open-access datasets. Despite considerable research efforts aimed at monitoring hypoxic states^22–25^, early diagnosis of pathological hypoxia^26–28^, and adapting oxygen therapy strategies^29–32^, most researchers do not make their corresponding datasets publicly available. There are a few publicly accessible datasets, such as the BIDMC^33,34^ and OSV^35,36^ datasets described in Table 1, which were derived from hospitals or low-altitude environments. However, due to issues such as scattered data, short sampling periods, and insufficient sampling rates, these datasets are unable to capture human performance at high altitudes, especially with varying altitudes. As a result, these publicly available datasets have limitations that make them unsuitable for various studies in high-altitude environments.

**Table 1:** A comparison of similar datasets with ours.

Dataset	Scenario	Participants	Data category	Continuity	Sampling rate	Duration of a single record	Total duration
BIDMC^33,34^	Hospital	Critically ill patients	PPG, Impedance respiratory signal	Continuous signals, Discrete values	125 *Hz*	8 *Min*	7 *H*
OSV^35,36^	Low altitude	Young and elderly people	*SpO*_2_	Discrete values	1 *Hz*	1 *H*	36 *H*
**Harespod**^44^	Hypobaric oxygen chamber (simulated high altitude)	Healthy young adults	PPG, Impedance respiratory signal, Heart rate, *SpO*_2_, Pulse rate	Continuous signals, Discrete values	100 *Hz*	50 *Min*	12 *H*

To address these issues, we present an open-access physiological dataset, named Harespod (High Altitude REspiration and SPO2 Dataset), that includes oxygen saturation, respiration, and their derived data in response to altitude changes on the plateau, along with key timestamps. The dataset covers an altitude range from 2 *km* to 4 *km*, ensuring various levels of altitude stress on the human body without significant health risks to most participants. With high sampling rates and continuous collection, we can obtain complete physiological signals during short-term altitude ascent. Through detailed annotations, even all the details of the physiological signals’ stress reactions to altitude changes can be understood. The stepwise ascent pattern of altitude enables alternating static and dynamic altitude stimulation to be captured in one go, thus expanding the dataset’s applicability and research value. This dataset is expected to facilitate the detection of human hypoxia status and improve the understanding of the delayed patterns of physiological signals at high altitude. Moreover, it could contribute to the development of effective and user-friendly health monitoring models and warning systems for outdoor workers at high altitudes settings. In situations with limited oxygen supply, this could prolong the duration of oxygen supply while ensuring the health status and providing better survival capabilities for outdoor workers at high altitudes.

## Methods

### Participant recruitment

All participants were recruited from college students through groups on social media platforms by the researchers. Students without prolonged highland residency experience were recruited from Northwestern Polytechnical University and confirmed to have no history of respiratory or circulatory diseases. Initially, a total of 23 participants (10 males and 13 females) between the ages of 23 and 31 were recruited. The purpose of the experiment, main procedures, considerations, and operational risks were explained to all volunteers in advance, and their verbal consent was obtained. Prior to the experiment, the researchers reiterated this information to each participant outside the experimental cabin, and written consent was confirmed. We provided an exit mechanism for all participants, allowing them to cancel or terminate the experiment at any time by notifying the researchers. During the experimental phase, if participants feel uncomfortable, the researchers in the hypobaric oxygen chamber will provide them with oxygen and immediately terminate the experiment. Simultaneously, researchers outside the chamber would promptly begin reducing the simulated altitude. The experiment was conducted between November 20, 2022, and March 28, 2023, with the consent of the participants. Respiration and oxygen saturation data were collected for each participant, lasting between 40 and 60 *min*. Key timestamps were recorded as participants reached different altitudes.

If there are missing key data types in the collected data, such as missing respiratory waveforms, they cannot be used for joint analysis. Moreover, if there are missing data for two or more altitude groupings out of the five altitude levels in the experimental design, it is not possible to reveal the pattern of physiological changes. In such cases, the participant’s data will be deemed invalid. Consequently, only data from 15 participants were utilized to construct the dataset. Summary information about the participants and dataset is included in Table 1.

The experiment involved collecting oxygen saturation and respiration data in a simulated high-altitude environment. All experimental procedures strictly adhered to relevant ethical regulations. The guidelines for human participants outlined in the Helsinki Declaration^37^ were strictly followed in all studies involving human subjects. Every effort was made to ensure the participants’ life, health, dignity, self-determination, and privacy. These data collection procedures received approval from the Northwestern Polytechnical University Medical and Laboratory Animal Ethics Committee, under the ethics review number: 202302035. Furthermore, the same ethics committee granted approval for the release of the oxygen saturation and respiration dataset in an open access format.

The protection of participants’ privacy is of paramount importance. As the study population is composed of graduate students, all participants share similar age, occupation, and lifestyle habits. These factors help to reduce individual differences among participants and minimize the need for data collection experiments to access participants’ private information. To ensure privacy, participant contact and anonymization are carried out by different individuals who do not communicate any of the private information. All of the above ensures that our dataset maximally respects the privacy rights of the participants.

### Experiment setup

As the proportion of oxygen molecules in the natural environment remains constant, simulating low pressure can create an environment similar to high altitudes with reduced oxygen levels. This induces a decrease in the amount of dissolved oxygen in the blood through gas exchange in the capillaries of the lungs, resulting in inadequate oxygen supply to the tissues. Consequently, respiratory patterns need to be adjusted to compensate for the lack of oxygen. In our study, we artificially generated high altitude conditions by lowering the air pressure, thereby eliciting physiological response signals in the human body. To produce varying degrees of response signals, we designed a reasonable altitude gradient. During each stage, detailed timestamps were recorded alongside the collection of data using physiological monitoring equipment. Each complete experiment lasted approximately two hours, with approximately 50 minutes of recorded data containing valid information. Prior to their participation, all subjects were fully informed about the experimental procedures, considerations, and risks involved. Additionally, a reasonable mechanism was implemented to ensure their voluntary withdrawal from the study if desired.

### Experimental environment setting

**Fig. 1:**
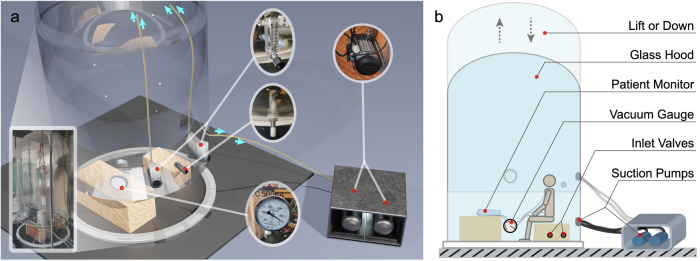
Schematics of the hypobaric oxygen chamber. (**a**) shows the composition and appearance of a more realistic hypobaric oxygen chamber, with some real photographs embedded. And the light blue arrows mark the position and direction of the gas as it flows in and out. (**b**) shows a participant performing an experiment in a hypobaric oxygen chamber, along with detailed labelling of the structure.

To create a simulated high-altitude environment, a hypobaric oxygen chamber was employed. This chamber consisted of two suction pumps (THOMAS, VTE 8, 6 *m*^3^/*h*, Germany) and a low-pressure-resistant glass hood (DYC 5000 A, China). Changes in pressure were monitored using a vacuum gauge (YiChuan, YNZ-100BF, -0.1-0 *MPa*, China), and fresh air exchange was facilitated through two inlet valves (BTAOO, 60 *L*/*min*, China) on the glass hood. The simulation environment is implemented indoors, where the temperature is regulated at 24 °C with an air conditioner. The actual temperature inside the chamber consistently remains between 24–26 °C. Throughout the experiment, the humidity in the chamber ranged from 53% to 58%. Participants could enter or exit the chamber via a pulley set located at the top of the enclosure. The design of the chamber closely resembles that in Fig. 1(a), which includes real photographs of the relevant components. Figure 1(b) provides a detailed labeling of the chamber’s components. Within the glass enclosure, a hose was employed to transfer air from the side bottom inlet valve to the top of the chamber, while the suction pump directly eliminated air from the side bottom. This setup ensured a constant flow of fresh air within the chamber and prevented the accumulation of excessive carbon dioxide. As indicated by the light blue arrows in Fig. 1(a), the airflow direction was directed towards the top of the glass enclosure, and then pumped out by the suction pump.

Sudden or constant fluctuations in altitude can make it arduous for the body to mount a timely response. A platform stage (or steady phase) is required to allow the body sufficient time to adapt to each alteration in altitude. To address this concern, we have developed a gradual altitude adjustment protocol. It is generally observed that individuals tend to experience plateau reactions from an altitude of around 2 *km*^38–41^, with more pronounced physiological stress reactions being observed at an altitude of 4 *km*^42,43^. Higher altitudes pose greater safety risks. Therefore, the altitude range was determined to be between 2 *km* and 4 *km*. Taking into consideration the acceptability of the subjects, the experiment should not be overly lengthy. We require a sufficient number of experimental groups to serve as mutual controls and to obtain abundant changes in stress response from them.

**Fig. 2:**
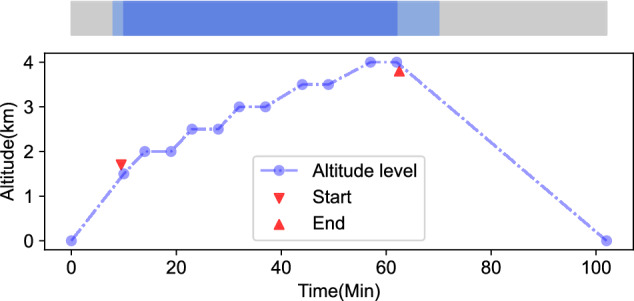
Altitude gradient adjustment schemes during the study and important time intervals containing key data.

Specifically, the pressure in the oxygen chamber was gradually reduced to simulate an altitude of 1.5 *km*. Physiological data was collected during this process. To acclimate the body to high altitude, a steady phase was maintained at an altitude of 2.0 *km* for 5 *min*. Subsequently, the simulated altitude was increased in increments of 500 meters, with a 5 *min* steady period allocated for each stage, reaching a maximum altitude of 4 *km*. The altitude simulation scheme is illustrated in Fig. 2, where the red triangles indicate the approximate start and end times of the data recording. The colored strip at the top of Fig. 2 represents the time period of our interest throughout the entire experiment. The grey strip depicts the duration of the entire experiment, the light blue strip represents the data collection process, and the deep blue strip corresponds to the data of our interest. Each experiment consists of approximately 40 to 54 *min* of data.

The oxygen proportion remains consistent in any open system, whether in highland or plain settings. In our case, the hypobaric oxygen chambers operate as semi-open systems and utilize suction pumps and inlet valves for gas exchange. However, the system does not significantly impact the oxygen proportion in the air. By adjusting the speed of air inflow and outflow, we can manipulate the absolute concentration of oxygen and maintain a specific simulated altitude. Before conducting the experiment, we examined the parameters of a hypobaric oxygen chamber that kept the simulated altitude at a specific elevation, based on negative pressure readings (vacuum degree).

To achieve this goal, we firstly adjusted the hypobaric oxygen chamber to a specific altitude. Subsequently, by adjusting the number of pumps and the turns of the intake valve, we changed the speed difference between air inflow and outflow. This indirect manipulation allowed us to alter the pressure conditions inside the oxygen chamber. During the experiment, we observed that slight changes in altitude usually appear in the vacuum gauge reading within 1–3 *min*. Therefore, if a particular combination of parameters can sustain the simulated altitude at a designated height for 10 *min*, we considered it to be a suitable parameter set and record it. Table 2 displays the gradient elevation regulation scheme and the optimal parameter combinations used to maintain altitude levels in the hypobaric oxygen chamber.

**Table 2:** Parameters for maintaining height in the hypobaric oxygen chamber.

Altitude (*km*)	Air pressure (*kPa*)	Vacuum level (*MPa*)	No. of pumps	Turns of inlet valve
2.0	78.9	0.0178	1	6
2.5	72.84	0.0238	1	4
3.0	67.24	0.0295	1	2
3.5	61.64	0.035	1	0.5
4.0	56.04	0.041	2	8

The quantity of pumps determines the rate of gas extraction from the hypobaric oxygen chamber (enhancing the negative pressure). And the turns of the inlet valve indicates the rate at which outside air enters the hypobaric oxygen chamber (resisting the negative pressure).

### Experimental procedure

Following the completion of exploratory tests, we successfully developed a standardized and reliable experimental operating procedure that allows for controllable simulation of high-altitude environments to stimulate the human body. Before the experiment, check the constituent components of the hypobaric oxygen chamber and ensure the proper functioning of the signal acquisition equipment. It is essential to maintain one inlet valve open throughout the procedure to facilitate fresh air exchange, while the initially closed valve can be adjusted as necessary during the experiment. The detailed protocol for conducting the experiments is provided below:

1. One researcher and the participant enter the hypobaric oxygen chamber together, while another researcher closes it by lowering the glass hood. Subsequently, the air pressure will be reduced by turning on a suction pump. The external researcher will closely monitor the negative pressure gauge reading at all times, confirming the simulated altitude in real-time and adjusting the parameters accordingly.
2. Once the simulated altitude approaches 1.5 *km*, the participant will be informed about the upcoming data collection phase and instructed to minimize physical activity. A client device, located outside the oxygen chamber, will receive the data stream from the monitor via Bluetooth, and all physiological signals will be recorded simultaneously.
3. Upon reaching a altitude of 2.0 *km*, the timestamp will be recorded and saved. Referring to the parameters in Table 2, a single pump will be used while adjusting the intake valve by 6 turns to maintain the current altitude for 5 *min*. After the stable phase concludes, the second air pump will be activated (as a double pump), and the simulated altitude will continue to rise.
4. While reaching 2.5 *km*, 3.0 *km*, and 3.5 *km*, their timestamps will be recorded separately. The external researcher will repeat the aforementioned actions, separately using parameter combinations of 4 turns & a single pump, 2 turns & a single pump, and 0.5 turns & a single pump, to maintain the altitude and collect physiological data at each corresponding altitude.
5. When achieving 4.0 *km*, the timestamp will be recorded and saved. Referring to the parameters in Table 2, double pumps will be used while adjusting the intake valve by 8 turns to maintain the current altitude for 5 *min*. Subsequently, one of the air pumps will be switched off (as a single pump) in order to reduce the simulated altitude. This marks the approximate end of the data collection phase. As the negative pressure approaches zero, the researchers inside the chamber can open the inlet valve without flow meter to accelerate the inflow of air, which is normally closed during the experiment.

### Physiological data collection

Oxygen saturation is the primary physiological parameter used to measure the participants’ hypoxic status in high-altitude scenarios. It is a discrete value derived from raw data collected over a short time period. Both the oxygen waveform and the resulting calculated oxygen saturation need to be recorded. These data were collected using photoplethysmography with a finger clip oximetry device (BerryMed, BSJ09001C, China) supplied with the BerryMed patient monitor (BerryMed, JHY-40, China). During the experiment, the finger clip sensor was worn on the middle or index finger of the participants’ left hand. The oximetry waveform data was sampled at a rate of 100 *Hz*, generating an oxygen saturation waveform with amplitudes ranging from 0 to 100 every 0.01 *s*. Simultaneously, the oxygen saturation and pulse rate values between 0 and 100 were calculated each second.

Alterations in respiration are triggered by the participant’s protective regulation of the body in response to changes in oxygen saturation status, indicating a shift in tissue oxygen demand. The modulation of respiration in response to hypoxic stimuli in the past period of time should be recorded. The signal was acquired using the impedance method with the ECG monitoring system provided by the BerryMed patient monitor (BerryMed, JHY-40, China). By observing the impedance variations between electrodes, it is possible to identify the amplitude changes in the chest and abdominal cavity caused by breathing movements. These electrodes should be worn with the assistance of the researcher inside the oxygen chamber. The respiratory waveform data was sampled at a rate of 100 *Hz*, generating a respiratory waveform amplitude between 0 and 250 every 0.01 *s*. Since the respiratory signal was collected through the ECG system, heart rate data between 0 and 250 *times*/*min* was simultaneously obtained.

All experimental data were collected using the BerryMed patient monitor, transmitted to the Windows client via Bluetooth, and locally saved in CSV (Comma Separated Value) format. Each raw data file consists of a timestamp column and a value column, with the timestamps accurate to the millisecond. Since the data category is already indicated in the file name, including column names within the file is unnecessary. The timestamps for reaching the specified simulated altitudes were recorded in a TXT file.

### Data preprocessing

To handle the high-frequency noise present in the original data, a low-pass filter was applied. The filter for respiration signals has an order of 8 and a critical frequency of 0.035, while the filter for oxygen saturation waves has an order of 8 and a critical frequency of 0.22. Redundant data was then removed by referring to the timestamp of the key interval (as shown in Fig. 2). The time of the altitude change event in the signal was identified by comparing the position of a specific waveform across different signals. Such processing facilitates a more dependable comparison and analysis of data at different altitude levels. The timestamps recorded by the researchers during the experiment for reaching the specified altitude supported this work. To minimize the impact of individual variances in tolerance to hypoxic conditions, all raw data was normalized.

## Data Records

All experimental data were saved in header-less CSV format and compressed using the 7z format (LZMA2 compression algorithm) to reduce file size. This allows for bandwidth conservation when accessing the data over a network. Both the raw data and cropped data, along with relevant records, were submitted to the Figshare data repository^44^.

**Fig. 3:**
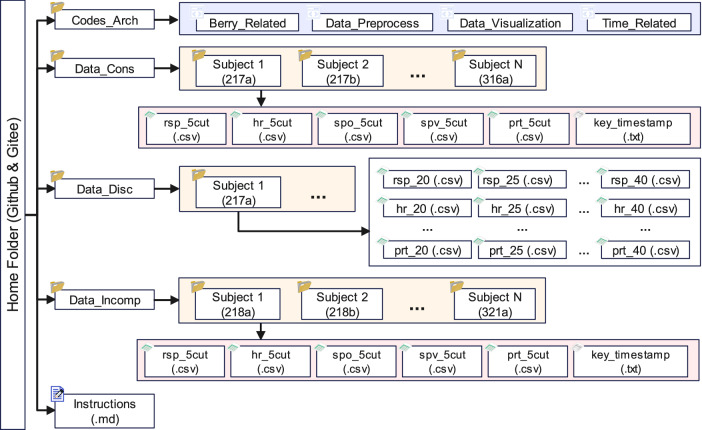
Main structure of the dataset folder. The main structure of the dataset folder is presented in the figure. The “Codes_Arch” folder contains the code for data acquisition, pre-processing, analysis and visualization. The “Data_Cons” folder stores the raw data collected from all participants in a consecutive manner. The “Data_Disc” folder contains the data divided based on the altitude levels. The “Data_Incomp” folder holds the incomplete data that has not been fully collected yet.

The main folder contains data folders, code folders, and an instruction document. Continuous raw data and segmented data were stored in the “Data_Cons” and “Data_Disc” folders, respectively. Each participant was assigned a folder named with a string of 4 characters (e.g., 217b) in length. The “Data_Cons” folder includes five physiological data files and one timestamp record (key_timestamp.txt) for each participant. The physiological data include respiratory waveform (rsp_5cut.csv), heart rate (hr_5cut.csv), oxygen saturation waveform (spo_5cut.csv), oxygen saturation values (spv_5cut.csv), and pulse rate (prt_5cut.csv). In the “Data_Disc” folder, each physiological data was divided into 5 segments based on altitude levels. For instance, respiratory waveforms were named rsp_20.csv, rsp_25.csv, rsp_30.csv, rsp_35.csv, and rsp_40.csv. All files contain typical time series data, including a column of timestamps and a column of values. The Markdown file in the main folder named instruction.md provides a basic description of the dataset and explains the role and cautions of each folder. The folder structure is illustrated in Fig. 3.

During the data collection process, which typically lasted for approximately an hour (and two hours or more for full experiments), some inevitable physical movements would corrupt the data. The incomplete data was stored in a folder named “Data_Incomp”, which still contained a significant amount of usable data. These data were also committed to the same master folder in both Github and Gitee repositories.

## Technical Validation

### Review of essential characteristics of dataset

**Fig. 4:**
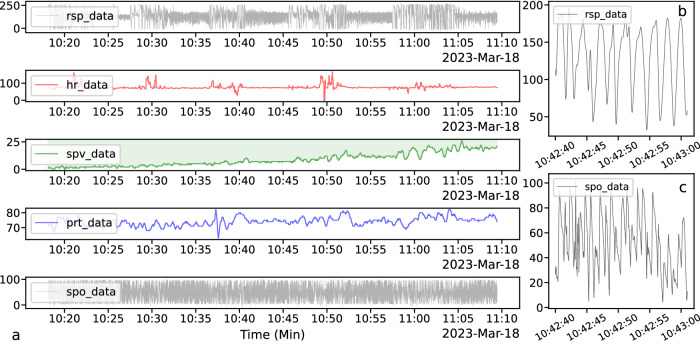
Presentation of Physiological Data of Participant 318c: (**a**) displays all types of data collected from participant 318 during the experiment. (**b**) depicts a partial respiration waveform of participant 318c, while (**c**) demonstrates a partial oxygen saturation waveform of participant 318c.

Initially, we examined the respiration data, oxygen saturation data, and other physiological parameters that were recorded simultaneously. Figure 4(a) displays all the data for participant 318c, while Fig. 4(b,c) show the detailed respiratory waveform and oxygen saturation waveform, respectively. To emphasize the similarity between oxygen saturation and pulse rate, the oxygen saturation curve was vertically flipped and filled with color to highlight the area over the curve, which represents its actual meaning.

**Fig. 5:**
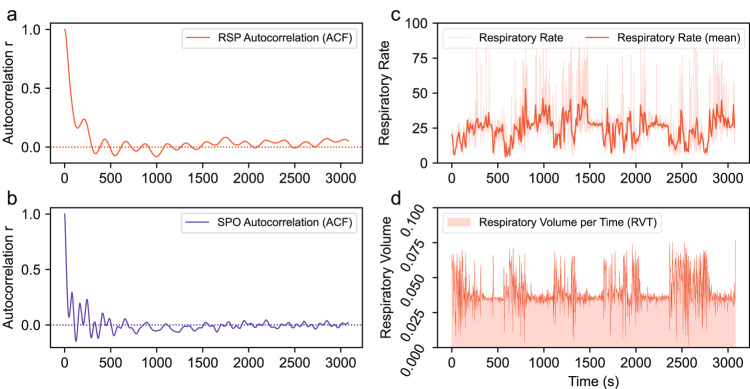
Several features of the raw respiration and oxygen saturation data obtained from participant 318c. Autocorrelation plots for the respiratory and oxygen saturation waveforms are presented in subfigures (**a,b**). Additionally, the respiration rate and respiration volume per time of the respiratory waveform are exhibited in subfigures (**c,d**).

The respiratory and oxygen saturation waveforms are inherently cyclical, and a stable physiological state causes them to exhibit a high autocorrelation. The autocorrelation of the respiratory and oxygen saturation waveforms for participant 318c were calculated and shown in Fig. 5(a,b). This implies that the change in altitude caused a change in the inherent periodicity of the physiological signal. The estimates of respiratory rate and respiratory volume were plotted in Fig. 5(c,d). The light red curve in Fig. 5(c) represents the estimated respiratory rate based on the original signal. It is evident that a large number of these estimates are unreasonable and not easily interpreted by humans. To address this issue, we calculated the average respiratory rate in units of 1000 data points (equivalent to 10 seconds) and replotted it in a manner more readable for humans, which is displayed as the red curve in the same subfigure. The light red area in Fig. 5(d) represents the estimated respiratory volume. We marked the boundaries with a red curve to facilitate the observation of respiratory volume fluctuations. In brief, both respiratory rate and respiratory volume show clear phases. During altitude changes, they exhibit large fluctuations, while they remain relatively stable during steady phases.

**Fig. 6:**
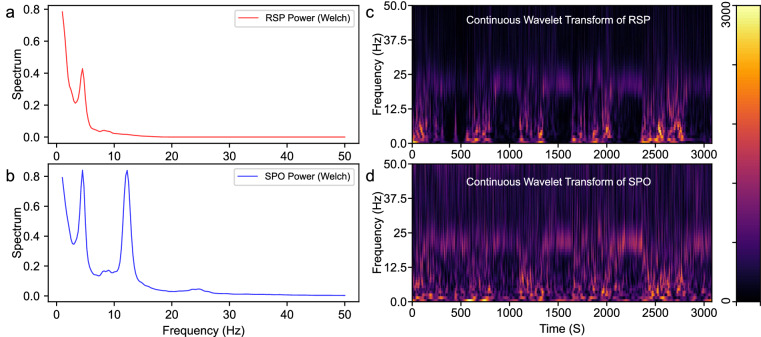
Spectral features of respiration and oxygen saturation of participant 318c. Subfigures (**a,b**) display the power spectra of respiration and oxygen saturation, respectively, while subfigures (**c,d**) show their time-frequency spectra.

Figure 6(a,b) show the power spectra of the respiratory and oxygen saturation signals, which depicts their energy distribution at different frequencies. This provides a reference frequency component for additional filtering operations and signal prediction. Figure 6(c,d) display the time-frequency spectra of these signals, showcasing the signal in both the time and frequency domains. The time-frequency spectra enables researchers to observe phases in the data (in temporal dimension) and identify the frequency range of interest (in frequency dimension).

### Distinguishing altitude levels with physiological signals

When individuals are at different altitudes, their physiological parameters tend to fluctuate significantly as a means of protective self-adjustment^45–49^. Therefore, these physiological parameters should demonstrate a discernible trend with increasing altitude.

**Fig. 7:**
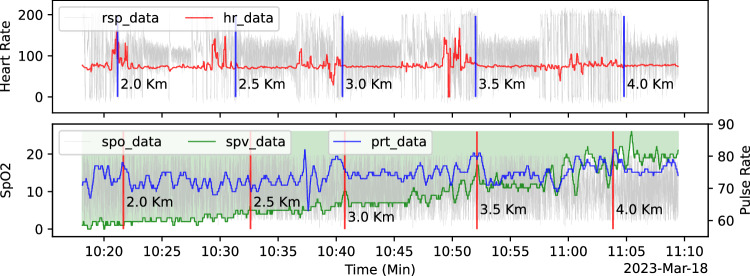
The events marker of respiration and oxygen saturation for participant 318c.

For each participant’s data, the actual times of events in response to altitude changes in the raw data can be annotated by referring to the timestamps in the “key_timestamp.txt” file. To illustrate with data from participant 318c, the respiratory and oxygen saturation signals’ response to changes in altitude were marked respectively with vertical lines of blue and red, as shown in Fig. 7. The data for different altitudes were split into separate files, which can be used to distinguish whether people are at different altitudes.

**Fig. 8:**
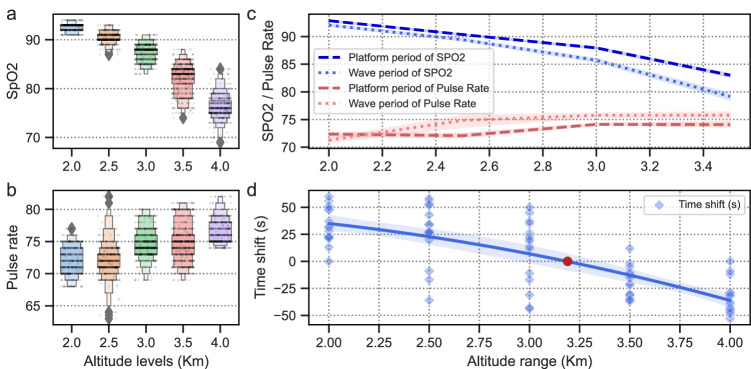
Oxygen saturation and pulse rate distribution of participant 318c at varying altitudes. (**a,b**) display differences in oxygen saturation and pulse rate for participant 318c across different altitude levels. (**c**) depicts differences in oxygen saturation and pulse rate for participant 318c across different altitude states. (**d**) demonstrates the difference between each participant’s respiration and oxygen saturation response time to each altitude. The red markers denote the altitude at which a transition in the delayed mode occurs.

Using the oxygen saturation and pulse rate data of participant 318c as an example, we divided the data into five groups based on altitude. Then we compared the oxygen saturation and pulse rate at different altitude levels using boxenplots (with seaborn). As illustrated in Fig. 8(a,b), we observed a clear stepwise pattern in their distribution at different altitudes. This indicates the reliability of our dataset in distinguishing between different altitudes.

### Detecting altitude change status with physiological signals

In certain scenarios, it is crucial to determine whether an individual’s current altitude is relatively stable or varies substantially. The effects of a stable altitude on the body remain relatively constant, whereas the effects of a changing altitude on the body are dynamic. The body must adapt to altitude changes in order to reach a state of homeostasis as quickly as possible, but such adaptation is not necessary at static altitudes. This implies that different altitude stimuli patterns induce distinct physiological signal features.

Although change point detection algorithms^50^ can be utilized to address similar issues, the presence of long transition periods and variable delays in the alteration of physiological signals on the plateau pose a challenge. Manual inspection remains the most accurate option and cannot be substituted. With the markers in Fig. 7, the cut-off points for the rising and steady phases of altitude were identified. The data for the different states were intercepted separately, and the corresponding characteristics were calculated. As shown in Fig. 8(c), we plotted the physiological characteristic curves for the different states as the altitude increased, using the oxygen saturation and pulse rate data from participant 318c.

By examining the steady and rising phases of oxygen saturation in the graph, it is evident that the rising phases at any altitude display lower oxygen saturation values than the steady phases, with the difference increasing as altitude rises. As for pulse rate, it was observed that a steady altitude of 2.5 *km* or higher was required to elicit an increase in human pulse rate, whereas rising altitudes affected pulse rate from 2.0 *km* or even earlier (which may be a compensatory effect). At other altitude levels, fluctuating states were consistently accompanied by higher pulse rates, and their difference remained stable.

### Response time of physiological signals to altitude changes

Before commencing calculations, it is typically necessary to specify the delay or lag of the signal. Joint estimation^51^, which depends on the dominant frequency of the signal (i.e., the length of the mean period), is currently the best available scheme for obtaining reasonable delay parameters and dimensions. However, the reliability of this approach remains uncertain when the delay spans multiple averaging periods. Our dataset provides timestamp files and other synchronized physiological signals that can assist researchers in annotating raw data more accurately.

Referring to the markings depicted in Fig. 7, the relative positions of the two markers can be leveraged to indicate the time difference between the altitude-induced responses in different physiological signals, given that all data share the same time axis. Specifically, the event timestamp of oxygen saturation (the red line) minus that of the respiratory waveform (the blue line) was used to denote the time difference (or time shift). Time differences were computed for each participant at each altitude, where positive numbers indicated later changes in oxygen saturation and negative numbers indicated earlier changes in oxygen saturation. These values were recorded in Table 3, with column names corresponding to each participant’s number, row names representing different altitude levels, and cell entries indicating the time difference (in seconds) between the respiratory and oxygen saturation responses at that altitude. A scatter plot was presented in Fig. 8(d) to facilitate observation of the distribution of time differences. A curve was fitted to the data, and the point at which it intersected with the horizontal line representing “Time Shift = 0” was marked with red symbols, facilitating the observation of the transition in delayed mode. Generally, the respiratory response was found to be faster at lower altitudes, while oxygen saturation exhibited a quicker response at altitudes above 3 *km*. However, due to significant inter-individual variability in response time differences, this outcome cannot be deemed rigorous.

**Table 3:** The difference between each participant’s respiration and oxygen saturation response time to each altitude.

Altitude (*km*)	Participants and time differences (*s*)														
	217a	218c	219a	223a	223b	310a	310b	314b	314c	318a	318b	318c	321a	328a	328b
2.0	56	13	23	37	21	60	48	23	0	48	51	31	28	22	32
2.5	34	56	53	−36	28	58	34	27	20	43	26	52	27	−9	−17
3.0	−44	35	−11	−31	14	45	37	26	23	12	21	19	50	−17	−43
3.5	−10	−22	−30	−32	−12	−31	−21	−36	−12	−2	−5	12	−15	−37	−35
4.0	−12	−9	−29	−45	−31	−40	−45	−46	−53	−48	−38	−43	−29	0	−32

Owing to these differences, patterns discerned from signals collected at low altitude environments may not be extrapolated to high altitude environments. Additionally, existing models and tools are not tailored to high altitude environments. Consequently, in high altitude scenarios, the use of models and tools developed based on low altitude data may lead to erroneous predictions of physiological parameter patterns. This highlights the importance of the dataset. Nonetheless, a comprehensive analysis of the data is necessary to yield more meaningful findings.

## Usage Notes

Anyone can access and use this dataset from the online data repository, provided that they comply with the relevant agreements and regulations.

The physiological data and timestamp records, obtained at designated simulated altitudes, were stored in CSV format and compressed in 7z format (using the LZMA2 compression algorithm). To decompress 7z files and access the CSV file, 7zip (https://www.7-zip.org) on Windows or p7zip (command line version of 7zip) on GNU/Linux can be utilized. CSV format has been the most widely used format for exchanging data between analysis software over the past few decades. It saves data as comma-separated values and can be viewed in any text editor such as Sublime Text with GUI (https://www.sublimetext.com) or Vim in CLI (https://www.vim.org). When viewing formatted data, a monospaced font such as “DejaVu Sans Mono” is recommended.

When processing the timestamp stored in the file, it is typically recognized as a text type. To analyze the data, a specific library is required to convert the timestamp into a date-time type. For instance, in the Python programming environment (Python Software Foundation, https://www.python.org/), the datetime (https://docs.python.org/3/library/datetime.html) library was utilized to parse the date-time format in advance. Alternatively, the read_csv function of the Pandas (https://pandas.pydata.org) library can be employed to automatically parse date-time data with the parse_dates parameter while reading the data. Data analysis can be conducted using the NeuroKit2^52^ (https://github.com/neuropsychology/NeuroKit) library, a popular neurophysiological signal processing toolkit.

When analyzing data, the following code assists users in parsing date-time information.


from datetime import datetimet_text_type = '2023-03-22 14:05:48.282't_datetime_type = datetime.strptime(t_text_type, '%Y-%m-%d %H:%M:%S.%f')import pandas as pddf = pd.read_csv('data.csv', index_col = 0, parse_dates = True, header = None)


The dataset presented in this study can serve as a valuable resource for the development of hypoxia detection algorithms. Along with the physiological data acquired during the experiment, timestamps of when the designated simulated altitude levels were reached are also provided. Using these timestamps, researchers or developers can review the original physiological data and segment it into appropriate fragments for specific research objectives. By incorporating detailed altitude information and oxygen saturation levels into the data snippets, more reliable features can be obtained, including breathing rate, breathing amplitude, respiratory volume, peak characteristics, phase characteristics, or complexity features. These features can be utilized to construct a classifier that distinguishes between the altitude and oxygen saturation levels associated with each signal segment using machine learning techniques. Researchers have the flexibility to modify the data segmentation strategy and adjust the model’s structure to achieve more analytical goals. For those who are not seeking an interpretable methodology, deep learning techniques^53,54^ offer a more straightforward solution for constructing a model. Furthermore, we recommend training the model using disturbed data from various participants and conducting leave-one-out testing on it.

The dataset can also be utilized for the development of hypoxia early warning systems. Researchers can directly integrate the aforementioned detection algorithm into their hardware systems and utilize the model’s detection results to guide subsequent operations. To achieve this, sensors similar to those used in our study should be included in the hardware system. It is not necessary for the sampling processes to be identical, but a similar data structure is required. In time-sensitive scenarios, models based on time series prediction can provide anticipated signal segments. By predicting signals through a predefined pipeline, developers can quickly understand the acquired signals. This provides system users with a brief period to take measures to combat the development of hypoxia. In extreme cases, such measures may even save lives.

The limitations of our dataset must be given sufficient attention. This dataset was collected in simulated high-altitude environments using a time-compact experimental protocol specifically designed to capture clear gradients and fluctuation features. However, this is not entirely equivalent to the physiological stress processes experienced in typical high-altitude travel, as those stimuli are usually gradual. In scenarios where the altitude changes too slowly, detecting underlying patterns in the signal becomes more challenging, while it is impractical to perform long-term continuous sampling without introducing additional variables. Models constructed based on our dataset can provide a reference research paradigm for this type of high-altitude change. Our dataset has a higher similarity to those datasets that have undergone rapid changes in altitude. Therefore, models based on our dataset can be directly used or minimally modified for such cases. Due to the difficulty in recruiting participants who met the inclusion criteria, and the need for data quality control, we ultimately obtained a small sample dataset consisting of only 15 participants’ physiological data. Given the inherent risks of high-altitude experiences, our study selected healthy young participants. Consequently, the results of related research cannot be directly applied to other populations with significant differences, such as children or the elderly. Data collection was carried out using PPG sensors and simplified ECG devices, which resemble commonly used practical devices. This characteristic makes it advantageous for applying related research results to the development of practical software and hardware systems.

## Data Availability

For the purpose of acquiring physiological data, the BerryMed patient monitor was employed. And the data export function was developed based on the C/C++ source code provided by the manufacturer. As there was no license from BerryMed to make their source code public, only the code we modified was provided. All physiological data is stored in header-less CSV format. To fully grasp the structure of the data file and integrate it into a specific project, please refer to the introduction provided in the Usage Notes section. Before analysis, the raw data underwent upsampling, filtering, and cropping procedures. For data collation and analysis, Python libraries like Pandas (Version 1.5.3), Scipy (Version 1.8.0), NeuroKit2 (Version 0.2.3), and Statsmodels (Version 0.14.0) were employed. Furthermore, Matplotlib (Version 3.7.0), Seaborn (Version 0.12.2) and NeuroKit2 were used for data visualization. To record the timestamp required to achieve the desired altitude during the experiment, the researcher employed a script program. The datetime library was utilized to acquire the timestamp and calculate the time periods. The source code is available on Github (https://github.com/oca-john/Harespod) and Gitee (https://gitee.com/oca-john/Harespod).
